# Crowdsourcing a Collective Sense of Place

**DOI:** 10.1371/journal.pone.0152932

**Published:** 2016-04-06

**Authors:** Andrew Jenkins, Arie Croitoru, Andrew T. Crooks, Anthony Stefanidis

**Affiliations:** 1 Department of Geography and GeoInformation Science George Mason University, Fairfax, Virginia, United States of America; 2 Center for Geospatial Intelligence George Mason University, Fairfax, Virginia, United States of America; 3 Department of Computational and Data Sciences George Mason University, Fairfax, Virginia, United States of America; University of Warwick, UNITED KINGDOM

## Abstract

Place can be generally defined as a location that has been assigned meaning through human experience, and as such it is of multidisciplinary scientific interest. Up to this point place has been studied primarily within the context of social sciences as a theoretical construct. The availability of large amounts of user-generated content, e.g. in the form of social media feeds or Wikipedia contributions, allows us for the first time to computationally analyze and quantify the shared meaning of place. By aggregating references to human activities within urban spaces we can observe the emergence of unique themes that characterize different locations, thus identifying places through their discernible sociocultural signatures. In this paper we present results from a novel quantitative approach to derive such sociocultural signatures from Twitter contributions and also from corresponding Wikipedia entries. By contrasting the two we show how particular thematic characteristics of places (referred to herein as platial themes) are emerging from such crowd-contributed content, allowing us to observe the meaning that the general public, either individually or collectively, is assigning to specific locations. Our approach leverages probabilistic topic modelling, semantic association, and spatial clustering to find locations are conveying a collective sense of place. Deriving and quantifying such meaning allows us to observe how people transform a location to a place and shape its characteristics.

## Introduction

The study of space and place has been the subject of multiple disciplines, ranging from geography [1,2] and planning [3,4], to health informatics [5], sociology [6,7], and psychology [8–10], to name but a few. Nevertheless, the concept of place itself remains rather elusive, as it is the outcome of cognitive processes that were up to now difficult to observe and curate. An often-cited definition has been provided by Tuan [2] who termed places as “…spatial locations that have been given meaning by human experience.” In the context of this paper, we consider place to be one’s perception of a location as it relates to one’s sociocultural views and personal experiences. Accordingly, places are formed through the reoccurrence of experiences and activities (of individuals or groups) at a certain location [11,12], which transform this location from a geometric concept (its three dimensional shape) to an experiential construct (conveying personal and/or public perceptions). Herein we refer to the defining characteristics of places, whether they are e.g. a financial district, a nightlife hotspot, or an artistic neighbourhood of a city, as platial signatures.

By its very nature, the concept of place is constantly evolving, as over time places are reconstituted with new meaning, reflecting for example urban dynamics [13], evolving sociocultural perceptions [14], or significant events [15]. While the assignment of a crisp geometric shape to places derived from social or collective meanings is often problematic due to vagueness and indeterminate boundaries [15,16], there exist substantial advantages to pursuing this effort: platial views offer new insights beyond traditional space perspectives as human activity is more aligned with place rather than geometric space.

The emergence of massive amounts of geotagged content (see e.g. [17]) is bringing forward a renewed focus on spatial humanities, or the study of human activities in, and in relation to, space and place [18]. We now have access to continuous streams of worldwide crowd-generated content, for example in the form of geotagged tweets and blog entries or Flickr and Instagram imagery. While it has been shown that such content can capture breaking events in the context of citizen journalism (see e.g. [19–22]) the question still remains of how well such crowd-contributed content can be mined to discover platial knowledge. The importance of developing new approaches to mining and reasoning with platial knowledge is evident [16,23,24] However, the types of data sources (i.e. geotagged media) available for mining platial knowledge present their own inherent challenges with respect to dimensionality, spatial and temporal coverage, linguistic style, etc. Thus the use of combined techniques from across multiple disciplines (i.e. geography, machine learning, social science) to study platial knowledge has proven most effective to date.

Enrico et al., [23] used a mixed approach, albeit with a single data source, that extracted spatiotemporal and semantic features from Twitter using a combination of spatial statistics, semantic modeling, and a neural network to detect human activity patterns from perceived platial content. Liu et al., [25] presented an approach to mining human representations of place and social activities from taxi trajectories and social media check-ins using a remote sensing framework to create social images. The images provide arguably land-use representations of platial knowledge and also highlight the fusion of multiple data sources to derive such information. Adams and McKenzie [26] evaluated platial content in travel blogs using topic modeling and spatiotemporal features to determine the thematic similarity between different places. This work highlights yet another dimension and data source of platial content, provided in this case, through tourist narratives at both an individual and collective event levels.

In this paper we are focusing on two distinct crowd-contributed sources: the consensus expressing crowd-curated Wikipedia content, and the uncurated content of individual tweets. While they are both expressions of crowd views they differ substantially in their purpose [27]. The former, i.e. Wikipedia entries, captures platial knowledge in the form of place entries, whose content is the outcome of a collaboratively-derived consensus on the main characteristics of such locations. Accordingly it can be viewed as an expression of the collective perception of such places. The latter, i.e. individual geotagged tweets made from such locations, simply express individuals’ concerns, observations, or interests while they are there. The research question that we pursue here is how to extract platial content from such crowd contributions, capturing the sociocultural characteristics of a place, and to quantify the alignment of these two sources with respect to platial content. Towards this goal we present an approach that leverages probabilistic topic modelling, semantic association, and spatial clustering to find locations of collective sense of place.

## Materials and Methods

In order to address our research question we proceed by collecting Wikipedia entries and semantic access statistics for various locations and Twitter streams originating from these locations as well. We then learn topical terms in such user-generated content, label them by assigning them into high-level categories, and compare them to quantify the level of thematic alignment between these two different sources. In this paper we present results from four major cities, namely New York City (NYC), Los Angeles (LA), Singapore (SG), and London (LDN). Furthermore, in order to highlight our research question at a finer geographical scale, we also present results from three areas within NYC (Lower Manhattan, Central Park, and Theatre District). We should note here that in the context of this paper we also use the term neighbourhood to refer to such sub-city level areas. We pursue our study at both scales because the notion of place is inherently multiscalar [28–30].

Regarding the sociocultural categories that characterize a place, we selected politics, business, education, recreation, sports, and entertainment as our high-level categories. These particular categories were chosen because they have been shown to be dominant in both Wikipedia and Twitter (see e.g. [31,32]), with entertainment in particular having a disproportionately strong presence in user-generated media [33,34]. Each of the above-listed high-level categories is an explicit category in Wikipedia and appears in over 50,000 articles. Previous studies have demonstrated content alignment with said categories for classifying trending topics [35], filtering tweets into new groups [36], and categorizing the professions of users [37].

**Fig 1:**
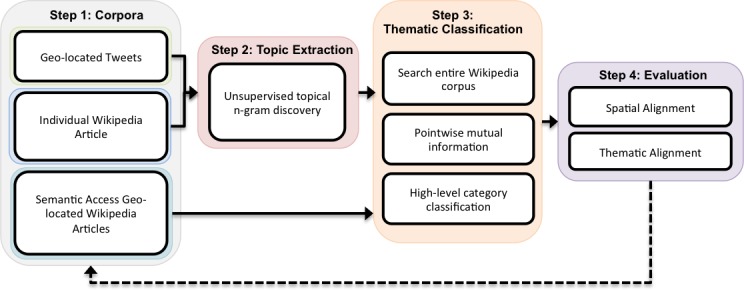
Flowchart describing overall process used to discover platial alignment. Step 1: sequential ingestion of Twitter, and Wikipedia article representing each spatial location, and all geo-located Wikipedia article titles spatially and semantic access statistics contained within the same location. Step 2: unsupervised topic discovery using topical *n*-grams. Step 3: determine the semantic overlap of topical *n*-grams and defined categories using Wikipedia search counts and PMI. Step 4: determine the statistical significance of each platial category and compare proportional alignment between Twitter and Wikipedia.

Our overall approach is presented in Fig 1, for which we briefly describe Twitter processing first. We collected geotagged tweets from our study areas using Twitter’s streaming Application Programming Interface (API) that returns a sample of 1% of all tweets. Previous studies have shown that such precisely geotagged tweets typically reflect a sample of approximately 2% [38] of the entire Twitter API traffic. For both the city and neighbourhood scales, Twitter data were gathered continuously for an entire month (April 2014), resulting in approximately 4.5 million tweets for NYC, 4.3 million for LA, 821,000 for SG, and 1.3 million for LDN. The tweets were filtered spatially using official geographic boundaries for all cities and neighbourhood areas [39–42]. Tweets were then grouped into 24hr time periods by location and processed using topical *n*-gram modelling.

In order to extract and label topics from our data corpus we applied a variation of the probabilistic topic model Latent Dirichlet Allocation (LDA, [43]), known as the topical *n*-gram model [44], treating 24-hour subsets of our data corpus as the equivalent of individual documents for the LDA analysis. To describe, LDA is an unsupervised generative probabilistic model that discovers latent structure in a collection of documents by representing each document as a mixture of latent topics (unigrams), where a topic is itself represented as a distribution of words that tend to co-occur [43]. In general, topic modelling enables the conversion of high-dimensional and noisy spaces of words and document allocations into a low-dimensional topic and document allocations. Blei et al. [43] described the use of LDA for dimensionality reduction for feature selection in supervised classifiers such as Support Vector Machine (SVM). In essences, topical n-gram model is applied for dimensionality reduction of tweets and Wikipedia articles.

Each detected bi-gram is considered part of a *topic of discussion*. Discussion topics may be classified linguistically as belonging to multiple high-level categories by considering their *Point-wise Mutual Information* (PMI) value [45]. PMI is a measure of association originating from information theory and statistics that calculates the probability of two events occurring given their individual and joint distributions. The measure has been applied in the field linguistics, from which we use here, to find associations between words using an online knowledge base. The probability of association is calculated by the collocation frequency of topical terms and categories. We thematically classify each topic as belonging to the high-level category that corresponds to its highest PMI value, which are estimated using the entire online Wikipedia corpus and comparing the frequencies of topic and category co-occurrences.

For our particular study, bi-grams perform better than unigrams or phrases, when it comes to their thematic classification. This is consistent with studies addressing the performance of *n*-gram classification in Twitter, which also supported the notion that bi-grams outperform other alternatives [46]. This is not surprising, as given the brief nature of tweets (at only 140 characters per tweet), unigrams are ambiguous, while meaningful longer phrases tend to be scarce. Based on the labelling of the bi-grams contained within them, individual tweets were classified under the 6 top-level categories as shown in Table 1.

**Table 1:** Total count of tweets per high-level category containing a semantically related topical n-gram.

	High-level Categories					
Cities	Politics	Education	Entertainment	Sports	Business	Recreation
NYC	12,893	27,122	242,226	34,998	32,060	55,832
LA	11,207	28,258	281,556	32,412	42,264	57,390
LDN	3,697	5,765	52,412	6,227	10,613	21,711
SG	2,207	5,717	28,863	3,621	6,247	7,363

Table 1 demonstrates the dominance of entertainment content in Twitter data. The results of this Table were obtained by setting a maximum value of 700 to the number of bi-gram topics that were extracted from our data corpus. In our studies we also compared the effect of varying the number of topics (i.e. from 100 to 700 in increments of 100) and found that the proportional allocations to high-level themes were consistent.

For single Wikipedia articles (Fig 1), we applied the same approach to analyzing individual article content, and more specifically the single entry for each city and neighbourhood that are subjects to our study. Each single Wikipedia entry was accessed programmatically using the article’s webpage address. We extracted topical bi-grams from these entries, and allowed for up to 30 topics per document, with up to 10 bi-grams per topic, reflecting the smaller size of these documents, compared to our Twitter data corpus. The number of topics was based on the empirical evidence from [47], as 30 was shown to optimize the Kullback-Leibler [48] divergence value. Bi-grams within each topic were thematically classified against each of the six high-level categories using the aforementioned PMI process.

We also investigate geo-located Wikipedia articles and their associated online semantic access statistics that are spatially contained with each study area. The rational for inclusion of this data is that Twitter is highly dynamic and event driven, and a single Wikipedia article (e.g. NYC), although derived from collective consensus, is comparatively more static. Hence, by thematically classifying each geo-located article using its title as the topic and the number of online page accesses from the same month as the categorical proportions, we extract a more dynamic representation of place from Wikipedia. We used DBPedia [49], which offers a more structured means to access Wikipedia content, to collect titles and geographic coordinates from geotagged articles [50]. The official boundaries of each study were used as before with Twitter to spatially filter the geotagged Wikipedia articles. We then used a Wikipedia statistics site [51] to gather the number of page accesses for each article title over the same time period as the Twitter data was collected (i.e. April 2014). As shown in Fig 1, the title of each article is processed using the same thematic classification approach as the topical n-grams. Instead of using topic counts to calculate category proportions (see Table 1), the number of page accesses was used. To illustrate, take for example the geo-tagged Wikipedia article entitled “Roseland Ballroom”, the article was thematically classified as entertainment and accessed 21,492 times in the month of April 2014. The process is repeated for all articles within the same boundaries and the total proportions for each category are calculated.

We selected a variation of LDA, topical *n*-gram model (i.e. bi-grams), given the short 140-character limit of tweets to achieve higher topic coherence. Individual bi-grams within each topic were programmatically sent to Wikipedia’s search engine to capture the search count (total number of articles containing the terms). This process was repeated for each high-level category and finally the joint count of each bi-gram and high-level category. We normalized each search count over the total number of articles in Wikipedia, which at the time of this study was N = 4,502,037. A PMI value was calculated for every bi-gram for each of the six high-level categories. The equation $PMI\left(w_{1},w_{2}\right)\;={\text{log}}_{2}\;\left(\frac{p\left(w1,w2\right)}{p\left(w1,\right)p\left(w2\right)}\right)$ depicts the PMI calculation adapted from [52]. The probability *p*(*w*_1_) is estimated by $f_{w_{1}}/N$, where is the Wikipedia web count of topic term *w*_1_ and *N* is total number of Wikipedia articles. The probability *p*(*w*_2_) is estimated by $f_{w_{2}}/N$, where $f_{w_{2}}$ is the Wikipedia web count of theme *w*_2_ and *N* is total number of Wikipedia articles. The probability of co-occurrence *p*(*w*1, *w*2) is calculated by $f_{w_{1}}f_{w_{2}}/N$, where $f_{w_{1}}f_{w_{2}}$ is the Wikipedia web count of *w*_1_ and *w*_2_, and *N* is the total number of Wikipedia articles.

In order to assess the PMI of the topical *n*-gram results related to the high-level categories we performed a manual assessment of a random subset of our Twitter data and found a nominal F_1_ score of 0.70. The bi-grams that Wikipedia could not disambiguate or interpret, thus resulting in a search count of zero, were not included in the results. This accuracy is consistent with similar studies using PMI, albeit without Twitter [53]. Recchia and Jones [54] compared PMI with Latent Semantic Analysis (LSA) using Wikipedia and Spearman rank correlation between human judgments of semantic similarity. These researchers reported correlations ranging between 0.73 and 0.86, and concluded that the simple PMI metric with large volumes of data correlates more closely with human semantic similarities ratings than more complex models.

Turning to the spatial aspect of our data, we accounted for the differences in scale and distance for which spatial clustering occurs at each location. Simply observing spatial processes using a single distance value across multiple locations will produce undesirable results as geographies vary considerably. As such, we used Global Moran’s I [55] to find an appropriate analysis distance per location (i.e. NYC) by repeatedly measuring spatial autocorrelation at incremented distances using the geo-located Tweets. Using the geotagged tweets and their PMI values per high-level category, we found the prominent distance with a statistically significant z-score (*z > 1*.*96*, *P < 0*.*05*), and detected the following measures: LA 701.3m, NYC 619.2m, SG 1,334.9m, LDN 731.4m. The distances were recorded for each of the four cities and parameterized as the fixed distance threshold in the local Getis-Ord Gi* [56] statistic along with the PMI values for each category.

The Getis-Ord Gi* statistic is used to discover local spatial clusters with high semantic values within the boundaries of each city. To briefly describe, Getis-Ord Gi* is a local spatial statistic that produces z-scores and p-values for each feature by looking at neighbouring features given a weighting, in this case distance is used as the weight, to determine spatial clusters of high or low values. We calculated the statistic by taking the sum of a single tweet PMI value combined with it’s neighbours within the set distance, and compared this to the expected local sum from all the tweet PMI values combined. When the difference between these two proportions is considerable and not by random chance, the single tweet is labelled as significant. A tweet with a high PMI similarity score is interesting, but may not be a statistically significant hot spot or a place with nearby features with similar place expressions. Moreover, a feature (i.e. single geotagged tweet) will have a high PMI similarity value and be surrounded by other features with high values as well. We determined statistical significance at the *P < 0*.*05* level and considered only z-scores greater than +1.96.

We selected the Renkonen [57], percentage similarity index (*PS = Σ min(p*_*1i*_, *p*_*2i*_)) to assess proportional alignment of high-level categories due to the minimal affect of sample size differences [58]. The similarity measure is traditionally used in measuring the proportional abundances of species between communities and habitats. We used the counts per high-level category to calculate percentages between each individual source at different locations (i.e. just Twitter or Wikipedia) and then a cross-source comparison (i.e. Twitter and Wikipedia). More specifically, the counts of topical terms per high-level category (e.g. Table 1) are expressed as percentages that sum to 100% for each location. Each location is compared to all other locations in pairwise steps using the Renkonen index. For instance, comparing the percentage similarity between NYC and LA entails taking the minimum percentage value between corresponding high-level categories (i.e. NYC politics vs. LA politics) and summing the values across all high-level categories. The higher the percentage value the more similar the places and conversely a lower value would indicate dissimilarity.

We then used non-metric multidimensional scaling (NMDS) to visualize the Renkonen similarities as dissimilarity using the rank-order of the data [59]. The combination of NMDS and Renkonen similarity has previously been used to investigate and visualize percentage similarities of habitat abundances [60] and categorical abundance [61]. Both works cite this approach as having low sensibility to a limited number of high-level categories, which makes this appropriate for our work. Given the Renkonen similarities values expressed between 0 and 1, we first converted these values to dissimilarities by subtracting each value by 1 to form our matrix. NMDS seeks an ordination of the data in which the distances between all pairs of data points, in this case the places from Twitter and Wikipedia, are at the greatest distance apart, while maintaining rank-order agreement with their dissimilarities. Meaning data pairs with dissimilarity less than other data pairs will have a shorter distance apart in the visualization from other data pairs with greater dissimilarity values. NMDS uses an iterative process to decrease the stress of rank-order agreement between distances and dissimilarity values; as such, we explored various iteration values to minimize the stress to a near 0 value as described in [59].

## Results

### Spatial alignment between Twitter hotspots and corresponding physical locations

**Fig 2:**
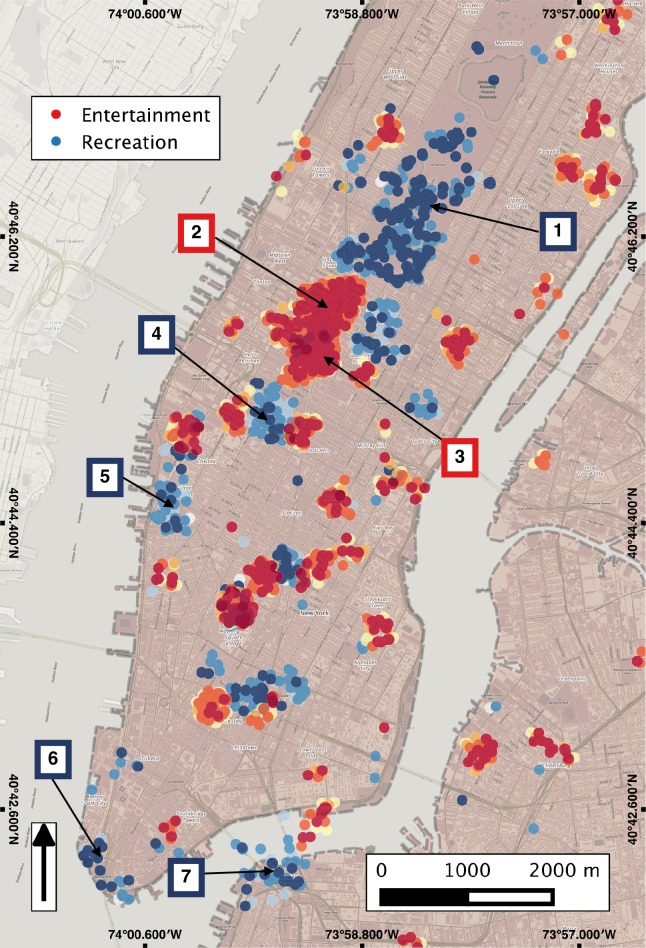
Statistically significant clusters of recreation and entertainment categories concentrated over Manhattan, NYC. For reference, the following sample places are labelled in the map with colour coded borders based on the legend features: (1) Central Park, (2) Broadway, (3) Times Square, (4) Madison Square Garden, (5) High Line Park, (6) Battery Park, and (7) Brooklyn Bridge Park.

In order to assess the degree to which Twitter content originating from various locations reflects the characteristics of these locations, we compare spatial clusters of thematic content in Twitter data to the corresponding physical neighbourhoods and their thematic characteristics as reflected in Wikipedia. Fig 2 shows the resulting high value clusters for two categories, namely entertainment (red) and recreation (blue) in a subset of Manhattan, NYC. In this Fig we show the spatial distribution in finer resolution in order to better communicate the level of alignment between Twitter clusters and corresponding physical places: green clusters align at, or near, known areas of recreational affordances such as parks and squares, with the most notable cluster spatially aligned with Central Park. Similarly, the entertainment clusters align with established local entertainment hubs such as Times Square, Madison Square Garden, and the most notable cluster being at Broadway in the Theatre Sub District, just southwest of Central Park.

**Fig 3:**
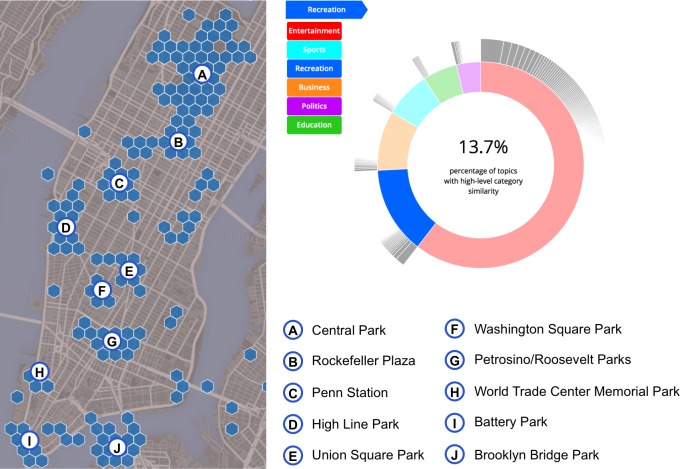
Spatially significant high value recreation clusters over NYC. The hexagon map depicts the statistically significant spatial clusters by point count coloured in blue. The results show a significant spatial alignment with prominent parks in NYC. The pie chart (right) shows that 13.7% of the total topics discovered from the entire NYC Twitter resulted in the highest semantic similarity with recreation out of the other five categories. Large clusters are annotated with location names to show spatial alignment.

Once these clusters are aggregated locally we have an even clearer view of this thematic alignment. These clusters are visualized in Fig 3, showing that the recreational clusters of Twitter content are overlapping not just with Central Park, but also with multiple parks throughout the city, including Battery Park, Brooklyn Bridge Park, High Line Park, etc. This supports the argument that at this scale of analysis Twitter content is reflective of the local platial characteristics. In the same figure, the pie chart on its right-hand side shows the relative portion of Twitter content that is considered as *recreation* within our Twitter data corpus for this area (namely 13.7%, second to the dominant *entertainment* category). Of course, as we see in Fig 3, we also receive few false positives (e.g. spots that are not aligned with parks in that figure). This is due to two reasons. First, there is some inherent fuzziness in the use of various terms, which may have multiple meanings in various contexts, yet are assigned their primary label in our clustering. For example, we often encounter the term *read* in tweets originating in the vicinity of airports, leading to the detection of local educational hotspots (as the term is primarily considered indicative of educational activities), even though in that case it is simply used by passengers who read books or periodicals while awaiting their plane. Second, the social media data may often indicate need rather than support for a certain type of activity. A small local square for example, may be serving as an informal recreational spot, even though formally it may not be recognized as such.

**Fig 4:**
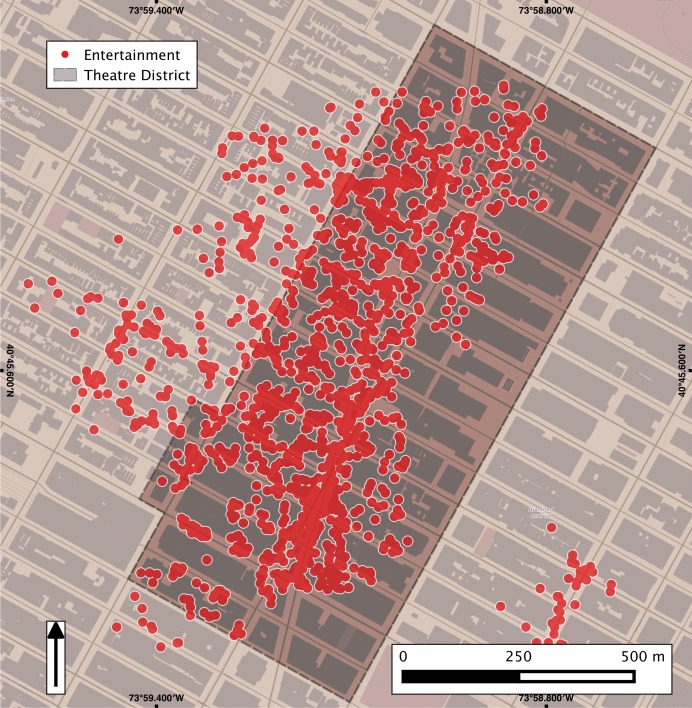
Depiction of spatially significant clusters and the Theatre Sub District, NYC. The spatial concentration of entertainment tweet clusters (red dots) contained within, and nearby, the Theatre Sub District (yellow polygon) show the alignment of platial expressions with local affordances (i.e. theatres around Broadway). We observe the dynamics of platial expressions with spaces adjacent to the official boundary being reconstituted with entertainment expressions.

While the above results show the overall alignment of thematic hotspots, we also compared the extent to which the crowdsourced spatial footprint of a thematic place aligns with its formally-defined extent. In Fig 4 we show the spatial extent of entertainment Twitter clusters (red dots) at the neighbourhood level for the Theatre Sub District, NYC. Beyond the spatial alignment, this Fig also illustrates how a place affordance extends beyond the formal boundary of that place due to human activity and/or expression: as the public moves to or from the Theatre District, it remains *immersed* in the thematic character of that place. As a result of this process, we have a collective reconfiguration of the platial boundary, shifting it westwards of its formal outline. Thus, social media content can capture places not just as the aggregate of their form and functions [15], but also by revealing the transitional immersion into the themes that these functions enable.

**Fig 5:**
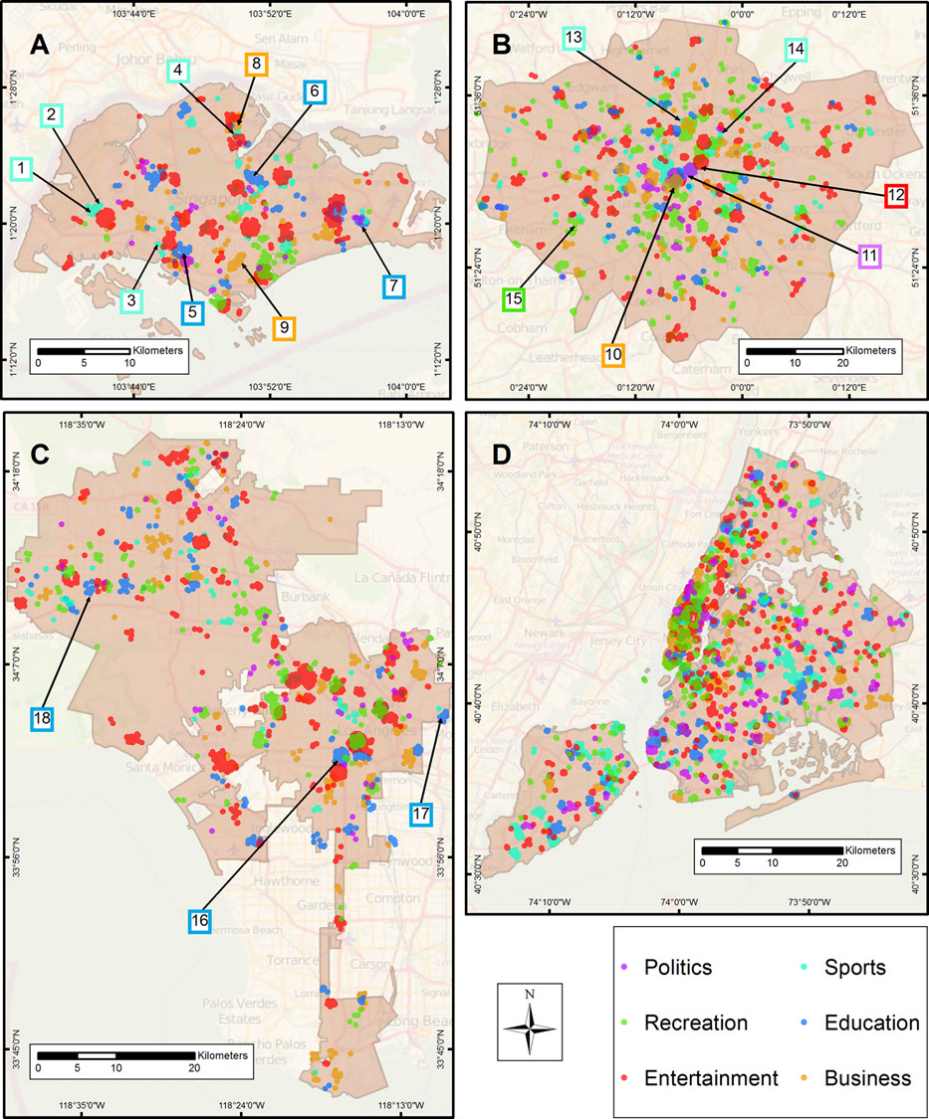
**Maps depict significant hotspots for each of the high-level categories for (A) Singapore, (B) London, (C) Los Angeles, and (D) New York City.** Additionally, the maps are annotated with sample locations were the hotspot aligns with a place with common meaning such as a school or stadium. The outlines of the numbered boxes correspond to the map legend colours. In map (A) Singapore, 1) Jurong West Stadium 2) Golazo Futsal Singapore 3) Clementi Stadium 4) Yishun Stadium 5) United World College of South East Asia 6) Nanyang Polytechnic 7) Temasek Polytechnic 8) Yishun District and 9) Orchard Road. In map (B) London, 10–12) West End of London 13) Emirates Stadium 14) Olympic Stadium and 15) Richmond Park. In map (C) Los Angeles, 16) University of Southern California 17) California State University Los Angeles and 18) California Lutheran University.

We ascertained similar spatial alignment in SG, LA, and LDN for business, education, and sports categories using the aforementioned methods. Fig 5 provides overview maps containing the statistically significant clusters per category. Here we briefly highlight some specific physical features in each area that have common affordances of either shared activities or experiences, to further demonstrate spatial alignment. In SG sports clusters were detected in the western portion of the city near Jurong West Stadium and the Golazo Futsal Singapore. Additional alignment was observed in the central part of the city near Clementi Stadium as well as to the north near Yishun Stadium. Majority of the education clusters were found near schools and universities as expected with notable locations as the United World College of South East Asia, Nanyang Polytechnic, and Temasek Polytechnic. Business clusters were detected in the Yishun district primarily in the central business district. Additional clusters are observed in the southern portion of the city near the Orchard Road location, which is a popular shopping area according to Wikipedia, and a large tourist attraction.

For LDN, we found overlapping thematic clusters of business, politics, and entertainment concentrated in the West End of London area. The location contains numerous government buildings, businesses, and theatres. We observed sports clusters near both Emirates and Olympic Stadiums, and also near restaurants and sports pubs. For recreation, our results indicated strong spatial alignment with parks and mostly notably LDN’s largest, Richmond Park. In LA, significant clusters in the business category were observed near outdoor and indoor malls, shopping centres, and restaurants. However, we must note that we did not find a significant business cluster in LA’s Financial District. Comparing our results against geographic data from the LA city government, we observed clustering in the education category near such example locations as the University of Southern California, California State University Los Angeles, and California Lutheran University.

To further investigate the relationship between thematic hotspots and corresponding locations of interest, we analyzed the Euclidean distance between thematic hotspots relating to education and sports and corresponding facilities in LA using average nearest neighbour and proximity analysis. We first used average nearest neighbour analysis to understand the spatial distribution of facilities and then calculated the proximity of thematic hotspot points to these locations. To briefly describe average nearest neighbour analysis, the process calculates the minimum distance between a point and its closest neighbour, and repeats the process for all points to derive a mean distance that is compared to an expected mean random nearest neighbour distance [62]. This process produces the average nearest neighbour distance and gives a measure as to whether the points are spatially clustered or dispersed. We then subsequently looked at the average proximity of tweets to said facilities to get a relative indication of the distance between the two sources.

In doing so, we focused on LA due to the accessibility of a GIS database, and chose education and sports because these are themes that have facilities with understood meanings. We first calculated an average nearest neighbour distance of 1298.6m for official sports facilities using LA city government data [40], and a nearest neighbour index of 0.43 and z-score of -16.67. With an index value less than 1 and a significant z-score, this result indicates the spatial distribution of sports facilities within the city are clustered. We then calculated the average proximity distance between all sports hotspot points to their nearest sports facility. We found that distance to have a mean value of 158.6 m, which is less then the average nearest neighbour distance and indicates an overall closeness in proximity between sports related tweets and facilities such as stadiums and athletic fields.

This also suggests that tweets conveying an athletic theme tend to originate in the vicinity of such facilities, conveying the platial nature of such locations. Accordingly, the mean distance of 158.6 m can be viewed as a measure of the extent of this particular theme’s immersion potential. Similarly, we calculated an average nearest neighbour distance among all schools, colleges, and universities in LA and found it to be 956.7 m with a nearest neighbour index of 0.75 and z-score of -10.13. As before, this result indicates the educational facilities are also spatially clustered within the city boundary. The corresponding average proximity between education-related tweets and the nearest educational facility had a mean value of 406.1 m. This pattern is consistent with the above-made observations regarding sports facilities and their relation to thematically-focused Twitter content.

### Thematic alignment between Twitter and Wikipedia at the neighbourhood level

Above we discussed the spatial alignment of Twitter thematic clusters with corresponding facilities and neighbourhoods to show how thematic hotspots tend to coincide with relevant physical locations and their characteristics. We now turn to assessing the thematic alignment among the uncurated content of individual tweets and the consensus expressing crowd-curated Wikipedia platial content. In that sense, each neighbourhood can be seen as having a sociocultural signature, expressing the ensemble of themes that assign it a particular character. Our objective is to compare such signatures as they emerge in Twitter traffic to the corresponding content as it is harvested from Wikipedia. We do so by studying the various percentages of different thematic categories as they are used to describe various neighbourhoods in Wikipedia and compare them to the corresponding percentages of Twitter traffic originating from these locations. In order to pursue this objective we present our results first at the neighbourhood level, and then aggregating up to the city level.

**Fig 6:**
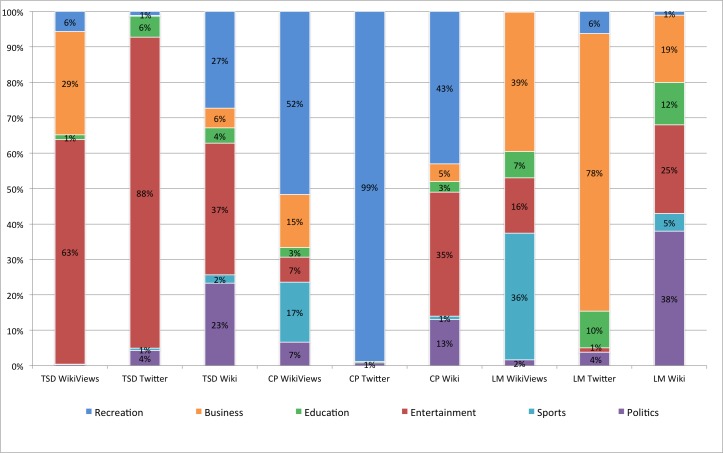
Percentages for each category, data source, and location in New York City neighbourhoods. The bar chart labels are presented in <Location> <Data Source> form. The locations are Theatre Sub District (TSD), Central Park (CP), Lower Manhattan District (LM). The data sources are Wikipedia Article Topics (Wiki), Spatial Twitter Topics (Twitter), and Wikipedia Semantic Accesses (WikiViews).

In order to highlight platial themes as they emerge at the *neighbourhood* level of analysis, we present results from NYC’s Theatre District, Central Park, and the Lower Manhattan Financial District in Fig 6. Each figure is a composite view of the proportions from Twitter and both Wikipedia topics and semantic page accesses by title. The outer pie chart on each graphic represents Wikipedia topics, the middle shows Twitter topics, and the inner depicts Wikipedia page accesses. In the Fig we have summarized the degree to which various themes express the particular nature of each neighbourhood, capturing for example the more recreational nature of Central Park and the more entertainment-oriented character of the Theatre District.

For the pair-wise comparison of such data we use the Renkonen index of similarity, whereby 100% similarity would indicate perfect thematic alignment, whereas 0% would reflect no alignment. Using this metric, the average similarity among the Twitter signatures (Fig 6) of the three locations was found to be 7%. The three locations have significantly low proportional alignment and thus indicate a uniqueness of each location amongst collective Twitter content. This is visually evident in Fig 6 with the Theatre Sub District comprised almost entirely of entertainment related content at 88%. The Theatre District contains numerous entertainment establishments, to include Broadway. Conversely, and as one would expect, 99% of the statistically significant content in Central Park is recreation related. As Central Park is one of the most well known parks in one of the largest cities in the world. For Lower Manhattan, we find business as the largest proportion at 78%, which is home to Wall Street and the most powerful financial district in the world. Ultimately, these findings highlight the differences in human experiences and activities at each location, and more importantly that unique expressions of place emerge from Twitter through uncurated processes.

We similarly assessed the single Wikipedia articles for each location (Fig 6). The average overall Renkonen similarity of this content for these three locations is 64%, which shows that single Wikipedia articles have higher proportional alignment and less uniqueness of place compared to Twitter. Central Park and the Theatre District resulted in the highest pairwise similarity of 87% compared to Lower Manhattan similarities of 49% (Central Park) and 57% (Theatre District). Considering the nature of Wikipedia entries as a reflection of crowd consensus, these similarity values can be viewed as an indicative reflection of the thematic variability among these locations. Regarding individual neighbourhoods, in the Theatre Sub District, entertainment is clearly dominant (37%), followed by recreation (27%). For Central Park, we find near the largest division between recreation (43%) and recreation (35%). In the Lower Manhattan District, home of City Hall and the financial district, proportions differ significantly from the other two locations with politics leading (38%), followed by entertainment (25%), and business (19%).

Additionally, we evaluated the Wikipedia titles with page accesses using Renkonen similarity from the percentages in Fig 6 and assessed the average overall similarity for these three locations at 40%. Thus indicating less proportional alignment and more uniqueness of place between the neighbourhoods. Although the uniqueness is not as distinct as Twitter at 7%, we do see a reduction in alignment compared to the individual Wikipedia articles taken from the same data source. Looking at the most prominent proportions in each neighbourhood from Fig 6; the Theatre District is 64% entertainment, Central Park 51% recreation, and Lower Manhattan 39% business followed by 35% sports.

**Fig 7:**
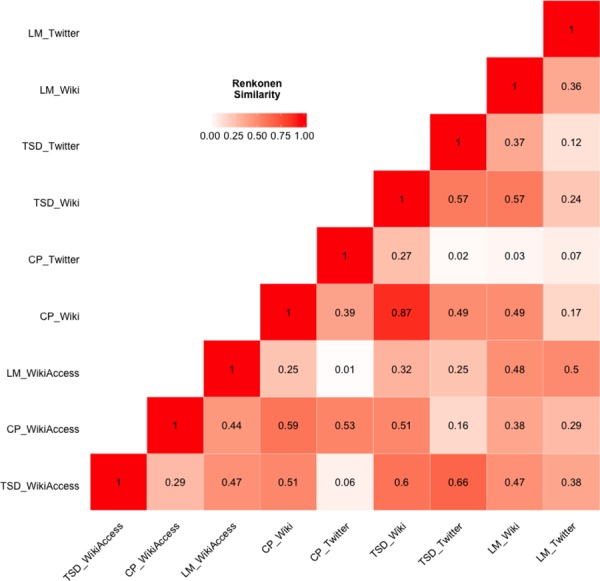
Renkonen similarity matrix for all three locations and data sources. The matrix schema shows a gradient between 1 (red) and 0 (white). The neighbourhoods are abbreviated as follows: Theatre District (TSD), Central Park (CP), and Lower Manhattan (LM). The data sources are differentiated as Twitter, single Wikipedia articles (Wiki), and geo-located Wikipedia article with semantic accesses (WikiAccess).

We compared the thematic content of Wikipedia (single articles and geo-located semantic access) and Twitter per location to assess proportional alignment by looking at Fig 7. Our findings show that for the Theatre District the highest similarity between Twitter and the geo-located article access at 66% followed by the single article and Twitter at 57%. The neighbourhood displays more pronounced particularities, namely its entertainment orientation. For Central Park, we have very high thematic alignment, compared to the other areas, between Twitter and Wikipedia (both single and geo-located page views) content (53% and 39% respectively).

Conversely, in the Lower Manhattan District, which is home to diverse affordances and therefore more multifaceted, the thematic alignment between Twitter and the neighbourhood single Wikipedia article was lower (36%). The comparison Twitter and geo-located page access resulted in higher alignment at 50%. Interestingly, our findings show the highest proportional alignment between two different neighbourhoods, Central Park and Theatre District, but using the single Wikipedia article for each area. This results points to homogeneity of Wikipedia, referring to its content and afforded activities, but also that these two neighbourhoods are spatially joined. Meaning the descriptions and expressions of place within Wikipedia for these two places share common semantics and lineages.

**Fig 8:**
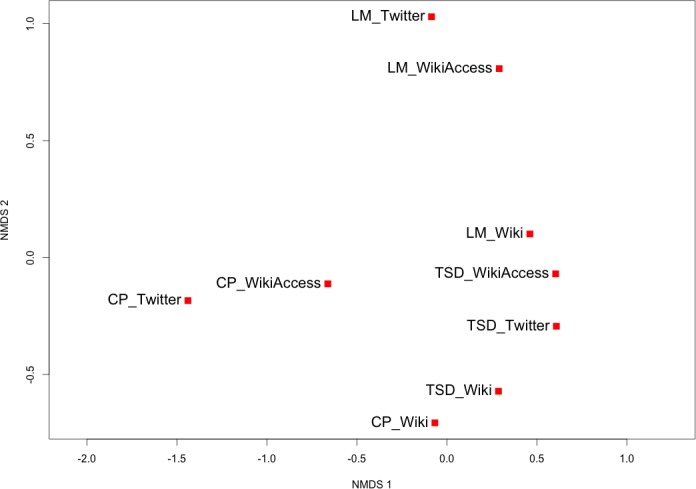
Non-metric multi-dimensional scaling of each neighbourhood and data source. The first and second dimensions of the ordination are represented with distances computed by rank-ordering between Renkonen dissimilarity values and distances in ordination space. The neighbourhoods are abbreviated as follows: Theatre District (TSD), Central Park (CP), and Lower Manhattan (LM). The data sources are differentiated as Twitter, single Wikipedia articles (Wiki), and geo-located Wikipedia article with semantic access (WikiAccess).

In Fig 8, we visualize the neighbourhoods using non-metric multidimensional scaling to better understand the proportional similarities. As previously discussed, the process of ordination uses the Renkonen dissimilarities derived from Fig 7. The distance between data points in Fig 8 is determined from the rank-ordering of dissimilarity values and distance values in ordination space for each pair of data points. From this we see two visual trends emerge from Fig 8. The first trend is the closeness of Twitter and Wikipedia semantic access proportions for each location, which indicates that at the neighbourhood scale a relative uniqueness of place, is revealed across these two different dynamic sources. Conversely, the second trend is the relative grouping of Wikipedia articles from different locations thus representing more homogeneity of the data source vice uniqueness of the places.

### Thematic alignment between Twitter and Wikipedia at the city level

**Fig 9:**
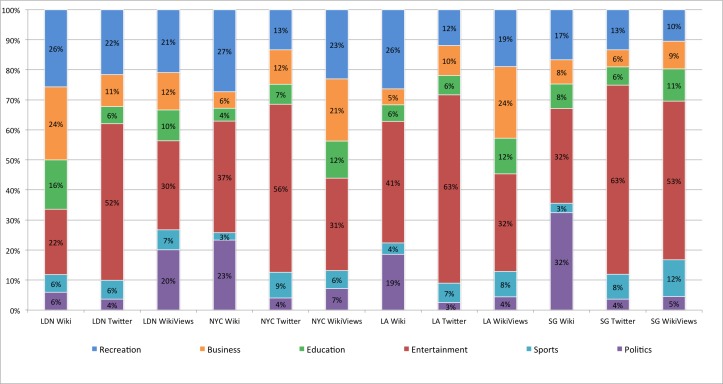
Percentages for each category and data source by city. The bar chart labels are presented in <Location> <Data Source> form. Locations include London (LDN), New York City (NYC), Los Angeles (LA), and Singapore (SG) and data sources are Wikipedia article topics (Wiki), spatial Twitter topics (Twitter), and geo-located Wikipedia semantic accesses (WikiViews).

**Fig 10:**
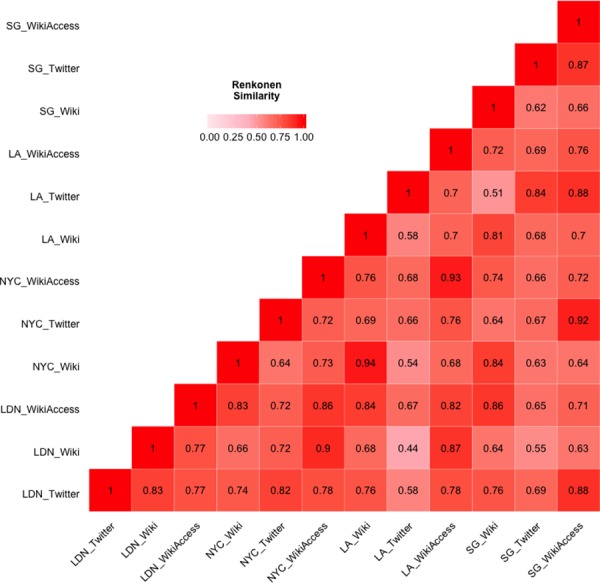
Renkonen similarity matrix for all three locations and data sources. The matrix schema shows a gradient between 1 (red) and 0 (white). The cities are abbreviated as follows: London (LDN), New York City (NYC), Los Angeles (LA), and Singapore (SG). The data sources are differentiated as Twitter, single Wikipedia articles (Wiki), and geo-located Wikipedia article with semantic accesses (WikiAccess).

We performed the same analysis at the city level, aggregating all results within the city boundaries, for NYC, LA, SG, and LDN. Focusing on Twitter, percentage data in Fig 9 and Renkonen similarities in Fig 10, our results show similar proportional distributions across the four cities with an overall average similarity of 69%. When compared to Twitter at the neighbourhood scale this result indicates a loss of locational uniqueness. This further suggests that aggregating social media content at the city level of analysis has a smoothing effect on thematic content, removing the fine level platial characteristics that we observed at the neighbourhood level. Moreover, one could make the argument that at this level of analysis the platial character dissipates, and we observe Twitter at large rather than particulars.

Comparing the Wikipedia articles (Figs 9 and 10) produced an average similarity of 76% across the four cities. Our findings show that NYC and LA have the highest pairwise similarity (Fig 10) of 94% with entertainment as the dominant category. The results for Wikipedia semantic accesses are somewhat alike the Wikipedia articles with an average similarity of 80%, with NYC and LA having 93% proportional alignment. Like our neighbourhood findings, we see strong similarity between Wikipedia articles and semantic accesses for different areas, pointing to the homogeneity of the source as opposed to locational distinction or scale.

**Fig 11:**
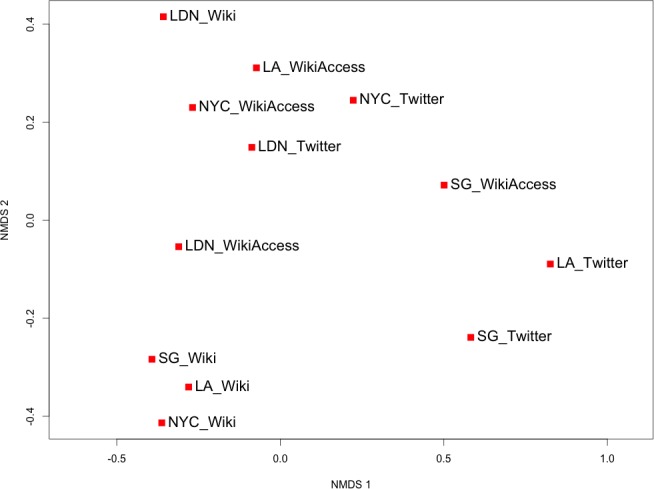
Non-metric multi-dimensional scaling of each city with data source. The first and second dimensions of the ordination are represented with distances computed using rank-ordering of Renkonen dissimilarities. The cities are abbreviated as follows: London (LDN), New York City (NYC), Los Angeles (LA), and Singapore (SG) The data sources are differentiated as Twitter, single Wikipedia articles (Wiki), and geo-located Wikipedia articles with semantic access (WikiAccess).

In Fig 11, we view the cities using non-metric multidimensional scaling as previously shown with the neighbourhoods to better understand the proportional similarities across the all data sources and cities. Once again, the Renkonen percentage similarities from Fig 10 are converted to dissimilarity values and visualized so that the data points are in rank-order agreement between distance and dissimilarity. As previously discussed, we explored various iteration values to minimize the stress or fit between distance and dissimilarity in the visualization. The apparent smoothing effect of scale and aggregation is further revealed at this level with the notable grouping of Wikipedia articles, minus LDN, and lack of clear groupings of Twitter and semantic accesses by location. The distances of dynamic content, Twitter and Wikipedia access, are tempered by the lack of a unique single category for any one location.

## Discussion

Our findings demonstrate how the meaning of place can be harvested through the analysis of crowd-generated content in the form of geotagged tweets. We do so by combining probabilistic topic modelling, semantic association, and spatial clustering to identify locations of collective sense of place. This approach also addresses the signal-to-noise problem found in Twitter, whereby, people may be in a place physically, but not necessarily participating or contributing to the shared meaning of a place. By contrasting such locations with the corresponding Wikipedia entries and semantic access we showed for the first time the thematic and spatial alignment between these two sources, supporting the argument that such content can be analyzed to reveal the shared meaning of place, as it emerges through human activities and perception.

Ultimately, the scale of place has a significant impact on its discernibility within sources as shown in our findings of neighbourhoods and cities. At the neighbourhood scale the particularities of place emerge, whereas zooming out to the city scale reveals more of the medium such as Twitter and Wikipedia instead of a particular location. Undoubtedly, the aggregation of sources at varying scales of analysis will produce different results, which is why we separately investigated cities and neighbourhoods. As our world is becoming increasingly urbanized and dynamic, gaining an understanding of the building blocks of these urban environments is bringing forth the need for a new science of cities [63]. The work that we presented here is contributing towards this goal, as it provides a new lens to observe platial content as it emerges from the people themselves, and allows us to do so at levels of spatial and temporal granularity that far exceed our past capabilities. The spectrum of applications that may benefit from such an understanding of space is broad, ranging from business (e.g. supporting location-based services) to security (e.g. detecting hotspots of unrest) and even health (e.g. studying health patterns not only as geometrical constructs, but also in association to underlying sociocultural data and attitudes). By moving from a geometrical view of the world around us to a platial view, we better support the quantitative study of the world’s character, in addition to its layout.
